# Prevalence of gastrointestinal symptoms in patients with influenza, clinical significance, and pathophysiology of human influenza viruses in faecal samples: what do we know?

**DOI:** 10.1186/s12985-015-0448-4

**Published:** 2015-12-12

**Authors:** Laetitia Minodier, Remi N. Charrel, Pierre-Emmanuel Ceccaldi, Sylvie van der Werf, Thierry Blanchon, Thomas Hanslik, Alessandra Falchi

**Affiliations:** EA 7310, laboratory of virology, University of Corsica-Inserm, 20250 Corte, France; Aix Marseille Université, IRD French Institute of Research for Development, INSERM U1207, EHESP French School of Public Health, EPV UMR_D 190 “Emergence des Pathologies Virales”, & IHU Méditerranée Infection, APHM Public Hospitals of Marseille, Marseille, France; Unité EPVO, Institut Pasteur, Paris-UMR CNRS 3569-Université Paris Diderot, Paris Sorbonne Cité, Cellule Pasteur, Paris, France; Unit of Molecular Genetics of RNA viruses, Institut Pasteur-UMR CNRS 3569-Université Paris Diderot-Sorbonne Paris Cité, Paris, France; Coordinating Center of the National Reference Center for influenza viruses, National Influenza Center (Northern-France), Institut Pasteur, Paris, France; Sorbonne Universités, UPMC Univ Paris 06, UMR_S 1136, Paris, France; INSERM, UMR_S 1136, Paris, France; Université Versailles Saint Quentin en Yvelines, UFR de Médecine Paris-Ile-de-France-Ouest, 9 boulevard d’Alembert, 78280 Guyancourt, France; Service de médecine interne, Hôpital Ambroise Paré, Assistance Publique-Hôpitaux de Paris, 92100 Boulogne Billancourt, France

**Keywords:** Influenza, Gastrointestinal symptoms, Stool, Respiratory infection, Intestine

## Abstract

This review provides for the first time an assessment of the current understanding about the occurrence and the clinical significance of gastrointestinal (GI) symptoms in influenza patients, and their correlation with the presence of human influenza viruses in stools of patients with confirmed influenza virus infection. Studies exploring how human influenza viruses spread to the patient’s GI tract after a primary respiratory infection have been summarized. We conducted a systematic search of published peer-reviewed literature up to June 2015 with regard to the above-mentioned aspects, focusing on human influenza viruses (A(H1N1), A(H1N1)pdm09, A(H3N2), and B). Forty-four studies were included in this systematic review and meta-analysis. The pooled prevalence of any digestive symptoms ranged from 30.9 % (95 % CI, 9.8 to 57.5; *I*^2^ = 97.5 %) for A(H1N1)pdm09 to 2.8 % (95 % CI, 0.6 to 6.5; *I*^2^ = 75.4 %) for A(H1N1). The pooled prevalence of influenza viruses in stool was 20.6 % (95 % CI, 8.9 to 35.5; *I*^2^ = 96.8 %), but their correlation with GI symptoms has rarely been explored. The presence of viral RNA in stools because of haematogenous dissemination to organs via infected lymphocytes is likely, but the potential to cause direct intestinal infection and faecal–oral transmission warrants further investigation. This review highlights the gaps in our knowledge, and the high degree of uncertainty about the prevalence and significance of GI symptoms in patients with influenza and their correlation with viral RNA positivity in stool because of the high level of heterogeneity among studies.

**Electronic supplementary material:**

The online version of this article (doi:10.1186/s12985-015-0448-4) contains supplementary material, which is available to authorized users.

## Background

The avian influenza A(H5N1) virus causes severe gastrointestinal (GI) symptoms and replicates in human intestinal tissues [1, 2], but the potential of human influenza viruses to cause direct intestinal injury during and/or after a respiratory infection remains unclear.

Although the main route of human influenza virus infection is respiratory, GI symptoms such as anorexia, diarrhea, vomiting, and abdominal pain are common manifestations [3–12], and may be a hallmark of severe influenza [13–18]. Seasonal and pandemic influenza viral RNA has been detected in stools of patients with confirmed influenza virus infection [19–27]. The influenza virus has occasionally been isolated from stool samples using cell culture [20, 22, 25].

The findings that faecal shedding of seasonal and pandemic influenza viruses could occur in patients with confirmed influenza raises the question of inadvertent human- human transmission, despite emphasis on droplet transmission and precautions for contact with respiratory secretions.

Our objective was to outline the current understanding about the occurrence and clinical significance of GI symptoms and human influenza viruses in the stools of patients with confirmed influenza virus infection. Knowledge from studies exploring how human influenza viruses spread to the patient’s GI tract after a primary respiratory infection has been summarized.

This review describes for the first time the current knowledge about the clinical significance and pathophysiology of human influenza virus in faecal samples, and, more importantly, highlights gaps in our knowledge and areas where research is warranted.

## Review

### Search strategy

Using PubMed, we searched the MEDLINE database for articles up to June 2015, without date restriction, using the terms ‘human influenza virus’, ‘faeces’, ‘diarrhea’, ‘stomach’, ‘faecal’, ‘intestinal cells’, ‘human intestinal’, ‘gut’, ‘viral load’, and ‘detection’. The terms were used alone or in combinations using Boolean operators. Only articles published in English were included and the search covered all years available in the MEDLINE database.

### Inclusion and exclusion criteria

We included studies according to the following eligibility criteria: (1) observational studies comparing clinical features between patients with influenza to estimate the prevalence of GI symptoms by influenza virus type and/or subtype; (2) case reports describing the occurrence of GI symptoms in patients with influenza; (3) observational studies regarding the detection and/or isolation of human influenza viruses in stools of patients with confirmed influenza by using upper and/or lower tract specimens; and (4) experimental studies on intestinal binding of human influenza viruses. The reference lists of all articles were reviewed for additional sources of data. All results were downloaded into an MS Word document and we searched for duplicate citations. Two investigators screened all articles by title and by abstract. All articles meeting the criteria were screened for information on study design, the time when the study was conducted, sample size, patient information, clinical presentation, nasopharyngeal and stool sample collection, number of virologically confirmed cases in nasopharyngeal specimens, influenza RNA detection and viral isolation from stool samples, method used for: viral detection, confirmation, and isolation.

### Article selection

**Fig. 1:**
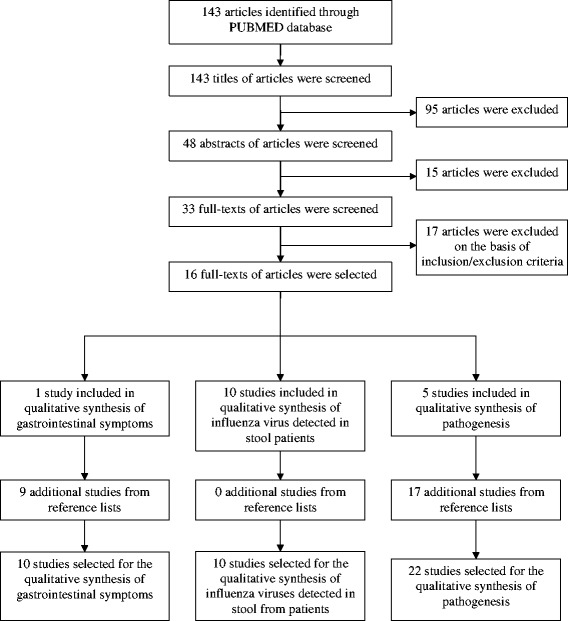
Flow diagram describing literature search and selection of studies

Our initial search yielded 143 articles, and after screening titles, abstracts and full-texts with inclusion criteria, 16 publications were selected. Twenty-six additional studies were retrieved by scanning the reference lists of articles selected (Fig. 1).

### Statistical analysis

We assessed the extent of the heterogeneity across studies of the prevalence of GI symptoms and the rate of detection of influenza virus in stools among influenza patients by using the *Q* statistic and *I*^2^ index [28]. These tests assessed whether variation across component studies was due to true heterogeneity or by chance. *Q* is distributed as a *χ*^2^ statistic and *I*^2^ describes the percentage of variation across studies that are due to heterogeneity rather than chance, with values ranging from 0 to 100 %. Data were pooled using either a random- or fixed-effects model depending on the degree of heterogeneity; acceptable heterogeneity was defined as *I*^2^ < 70 % [28]. In studies with a high level of heterogeneity (*I*^2^ > 70 %), a random-effects model was used. Statistical analyses were performed using MedCalc for Windows, version 12.5 (MedCalc Software, Ostend, Belgium).

### What is the occurrence of gastrointestinal symptoms in influenza patients?

We identified ten observational studies (six prospective and four retrospective) describing and comparing the occurrence of GI manifestation among patient mainly using reference methods: RT-PCR [6–12] and/or cell culture [4, 7] or in few studies, using serological tests and MDCK culture [3] or PCR [5] to confirm human influenza virus infection (see Additional file 1). Among the ten studies, four [3–5, 9] compared the clinical features between patients with type A and/or B seasonal influenza virus infection (see Additional file 1) and six compared the clinical features between patients with influenza A(H1N1)pdm09 virus and type A and/or B seasonal influenza virus infections (see Additional file 1).

As detailed in additional file 1, of the ten studies selected, four measured the occurrence of GI manifestations as ‘digestive symptoms’ or ‘gastrointestinal symptoms or disorders’ [3, 4, 7, 8] and six reported ‘vomiting and/or nausea and/or diarrhea and/or abdominal pain’ [5, 6, 9–12, 25]. Other symptoms such as heartburn and anorexia are not addressed in the studies included in this review.

Three of the ten studies reported that GI manifestations occurred more frequently in patients with confirmed influenza B virus infection than in those with confirmed infection with human influenza A (A(H1N1), A(H1N1)pdm09, and A(H3N2)) virus infection [3, 4, 6], in contrast to reports of similar occurrences of these symptoms among patients with seasonal influenza A or B [5, 8, 10]. The occurrence of GI manifestations among patients infected with influenza A(H1N1)pdm09 viruses has been reported to be higher than that in patients infected with seasonal influenza A viruses in three studies [7, 11, 12]. GI symptoms were more frequent among patients infected with influenza A(H3N2) than among those infected with influenza B viruses [9].

**Fig. 2:**
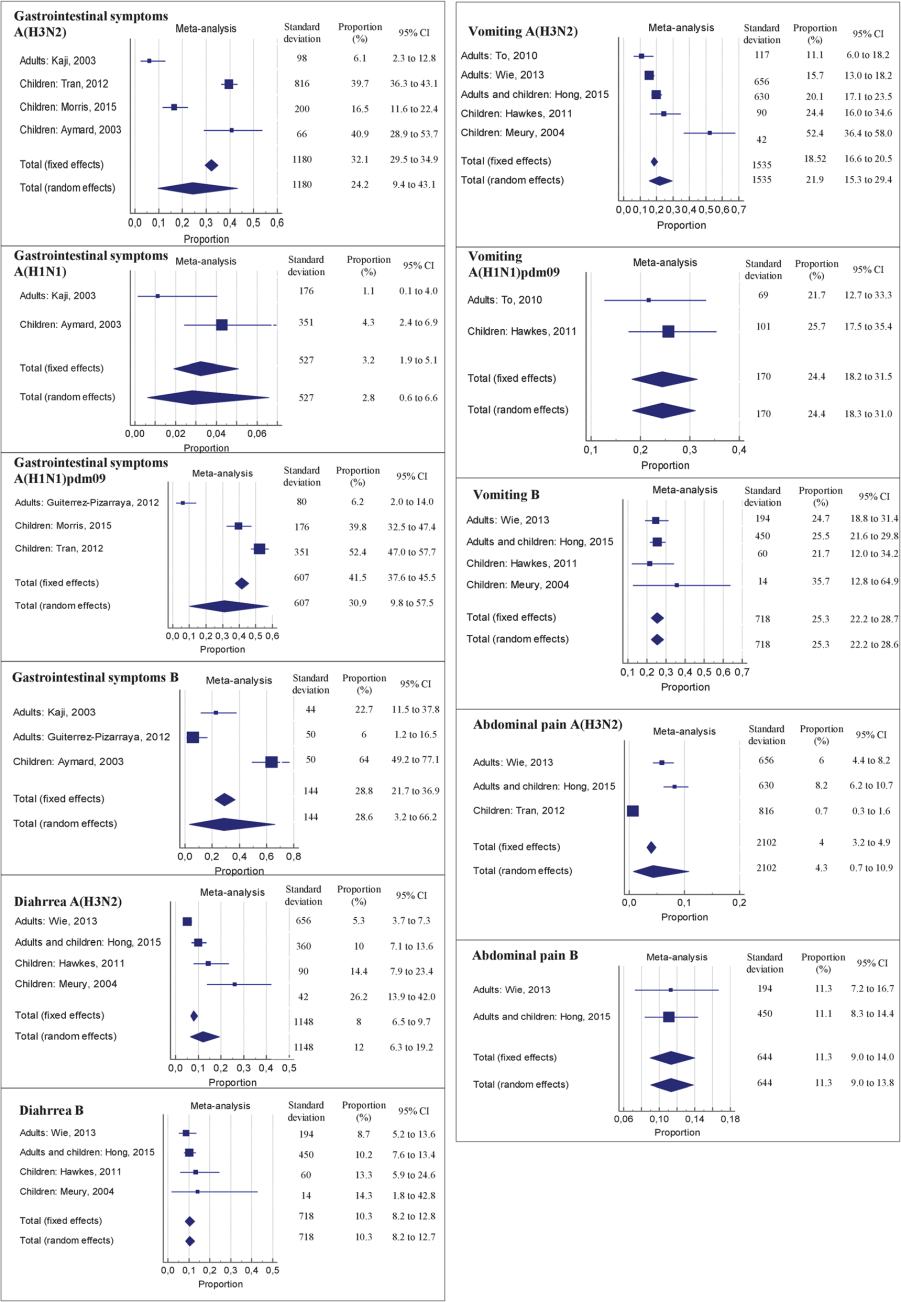
Forestplot of the meta-analysis on prevalence of gastrointestinal symptoms and pooled proportion of all types of gastrointestinal symptoms by virus type and subtype

Because the findings of the ten studies are apparently contradictory, we estimated the pooled prevalence of GI symptoms (‘digestive symptoms, or gastrointestinal symptoms or disorders’; ‘vomiting/nausea’; ‘diarrhea’ and ‘abdominal pain’) by a meta-analysis of the types and/or subtypes of influenza virus infections (Table 1; Fig. 2). As shown in Fig. 2, the results of the meta-analysis assessing the pooled prevalence of any gastrointestinal symptoms ranged from 30.9 % (95 % CI, 9.8 to 57.5; *I*^2^ = 97.5 %) for influenza virus A(H1N1)pdm09 infection to 2.8 % (95 % CI, 0.6 to 6.6; *I*^2^ = 75.4 %) for influenza virus A(H1N1) infection. The most commonly reported individual symptom was vomiting, with pooled proportions ranging from 25.3 % (95 % CI, 22.2 to 28.6; *I*^2^ = 0 %) for influenza B virus infection to 21.9 % (95 % CI, 15.2 to 29.4; *I*^2^ = 88.3 %) for influenza A(H3N2) virus infection.

**Table 1:** Heterogeneity of the meta-analysis on prevalence of gastrointestinal symptoms by symptoms category and virus (sub)-type

*Meta-analysis*	*Influenza virus (sub)-type*	*Number of studies*	*I* ^*2*^ *Statistic[IC]*	*Q Statistic (p-value)*
Gastrointestinal symptoms	A(H3N2)	4	96.9 % [94.4–98.3]	96.2 (p < 0.0001)
	A(H1N1)	2	75.4 % [0.0–94.4]	4.1 (*p* = 0.0439)
	A(H1N1)pdm09	3	97.5 % [95.1–98.7]	78.8 (*p* < 0.0001)
	B	3	95.6 % [90.4–97.9]	45.5 (*p* < 0.0001)
Vomiting	A(H3N2)	5	88.3 % [75.3–94.5]	34.2 (*p* < 0.0001)
	A(H1N1)pdm09	2	75.4 % [0.0–94.4]	4.1 (*p* = 0.0439)
	B	4	0 % [0–81.6]	2.5 (*p* = 0.4748)
Diarrhea	A(H3N2)	4	86.9 % [68.4–94.6]	22.9 (*p* < 0.0001)
	B	4	0 % [0.00–76.6]	1.7 (*p* = 0.6462)
Abdominal pain	A(H3N2)	3	97.1 % [94.2–98.5]	68.5 (*p* < 0.0001)
	B	2	0 % [0.00–0.00]	0.01 (*p* = 0.9005)

As shown in Table 2, the results of the meta-analysis assessing the pooled prevalence of any gastrointestinal symptoms in the children population ranged from 46.3 % (95 % CI, 34.2 to 58.7; *I*^2^ = 96.7 %) for influenza virus A(H1N1)pdm09 infection to 31.6 % (95 % CI, 36.3 to 43.1; *I*^2^ = 95.5 %) for influenza virus A(H3N2) infection. The most commonly reported individual symptom was vomiting, with pooled proportions ranging from 37.5 % (95 % CI, 13.5 to 65.4; *I*^2^ = 89.7 %) for influenza A(H3N2) virus to 25.6 % (95 % CI, 9.6 to 32.1; *I*^2^ = 60.9 %) for influenza B virus infection. Overall, heterogeneity across studies and/or wide confidence intervals were observed for the pooled prevalence of all types of GI symptoms analysed (Table 1, Fig. 2 and Table 2).

**Table 2:** Heterogeneity of the meta-analysis on prevalence of gastrointestinal symptoms by symptoms category and virus (sub)-type for the children population

*Meta-analysis*	*Influenza virus (sub)-type*	*Number of studies*	*Prevalence*	*I* ^*2*^ *Statistic[IC]*	*Q Statistic (p-value)*
Gastrointestinal symptoms	A(H3N2)	3	31.6 [36.33–43.15]	95.55 % [90.27–97.98]	45.1 (*p* < 0.0001)
	A(H1N1)	1	/		
	A(H1N1)pdm09	2	46.3 [34.24–58.7]	96.75 % [47.69–96.64]	7.54(*p* = 0.0060)
	B	1	/		
Vomiting	A(H3N2)	2	37.5 [13.54–65.36]	89.70 % [61.9–97.21]	9.7 (*p* = 0.0018)
	A(H1N1)pdm09	1	/		
	B	2	25.6 [14.7–38.35]	19.27 % [0–0]	1.23(*p* = 0.2657)
Diarrhea	A(H3N2)	2	19.6 [9.61–32.13]	60.97 % [0–90.93]	2.57 (*p* = 0.1095)
	B	2	14.42 [7.48–23.52]	0 % [0.00–0.00]	0.06 (*p* = 0.8010)
Abdominal pain	A(H3N2)	1	/		
	B	1	/		

### GI symptoms reported in case of severe influenza disease

Influenza A virus might induce severe GI complications. Several studies reported GI symptoms such as acute appendicitis, abdominal pain [17], and haemorrhagic gastritis [14, 29, 30] in patients with severe influenza, especially among children. The studies are described below.

Development of severe abdominal pain including appendicitis [16] or haemorrhagic gastritis of varying severity after a typical influenza-like illness was reported in children with influenza virus infection [14, 16, 29, 30]. During the A(H1N1)pdm09 pandemic clinicians reports noted an increased incidence/severity of acute appendicitis [31]. Some influenza A(H1N1)pdm09 cases of appendicitis have been reported in the literature [13, 31, 32] but in all these cases it is difficult to prove whether the A(H1N1)pdm09 virus infection caused the appendicitis or whether it allowed the occurrence of bacterial secondary infection. Cases of influenza A(H1N1)pdm09 virus infection mimicking acute abdominal pain in pregnancy [18] and a case of haemorrhagic colitis after A(H3N2) influenza virus infection in a 21-year-old man have been described [33].

Two cases of sudden fatal influenza B virus infection in young children complaining of abdominal pain and vomiting without influenza-like symptoms have been reported [17]. During the 2003–2004 influenza season, 153 deaths of children due to influenza (98 % influenza A) were reported in the United States, of which 39 % presented with vomiting, and 6 % with vomiting in the absence of respiratory symptoms [15].

Overall these GI complications could have several aetiologies as could be associated with oral administration of oseltamivir for the treatment of influenza [34], to non-steroidal anti-inflammatory use, to a direct viral effect or to a bacterial secondary infection. Precise understanding of intestinal complications as a consequence of an influenza virus infection could be drawn from in situ hybridization or PCR of infected tissues. The mechanisms of severe GI complications during an influenza virus infection need to be elucidated and clinicians should be alerted to the possibility of an increased incidence/severity of GI symptoms in patients with influenza.

### Occurrence of influenza virus in the stools of children and adults infected by influenza virus

**Fig. 3:**
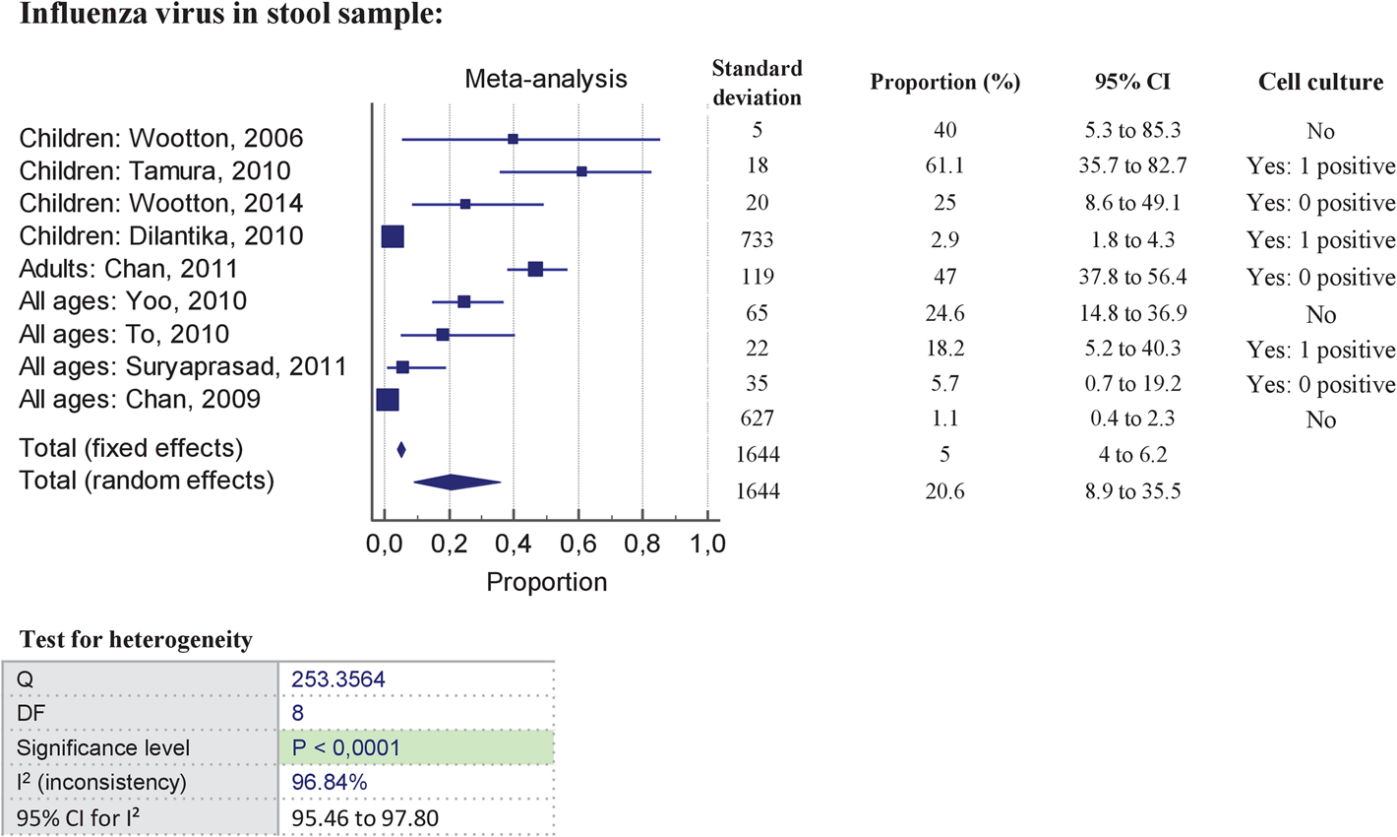
Forestplot of meta-analysis on prevalence of influenza virus detection on stool samples, pooled prevalence and Q test and I^2^ test

There are ten reports of faecal viral RNA shedding after analysis of: (i) hospitalized adult and/ or paediatric patients [19–25], or not hospitalized [26], (ii) adult patients with diarrhea enrolled by general practitioners [35] and (iii) patients of various ages in a retrospective analysis on the aetiology of gastroenteritis performed using stool specimens collected in a previous study [27]. The prevalence of influenza viral RNA in stool, detected by RT-PCR in all included studies, ranged from 3 [22] to 71 % in studies on children, from 7.2 % [27] to 47 % [23] in studies on adults, and from 0.06 [26] to 44 % [25] in studies on patients of various ages. Figure 3 shows the results of the meta-analysis, which assessed the pooled prevalence of influenza viral RNA in stools at 20.6 % (95 % CI, 8.9 to 35.5; *I*^2^ = 96.8 %). Among the ten studies reporting faecal viral RNA in stools, six of them have conducted cell culture experiments viral RNA stool [20–23, 25, 26], detected in all included studies by RT-PCR, and three of them described one positive culture [20, 22, 25] (Fig. 3). Studies are detailed in additional file 2.viral RNA Among the ten studies reporting faecal viral RNA in stools, six have conducted cell culture and three have described one positive culture (Fig. 3).

### Pathogenesis

While influenza virus is likely to spread to the GI tract of patients after a primary respiratory infection, the route of dissemination remains unknown. Current knowledge explains the detection of human influenza viruses in faeces because of: (i) swallowing of influenza viruses from the upper respiratory tract; (ii) remnants of infected submucosal intestinal antigen-presenting immune cells; and (iii) virus replication in intestinal cells.

### Swallowing of Influenza viruses from the upper respiratory tract

A low pH environment, such as in the stomach, should render most influenza viruses non-infectious by inducing an irreversible conformational change of the viral haemagglutinin [36]. For this reason, influenza virus detection in stools has generally been attributed to swallowed respiratory secretions. However, in this case the acid lability of influenza viruses that destroys their infectivity when passing through the stomach could also result in viral RNA degradation. Therefore, in the hypothetical scenario of swallowed influenza viruses from the upper respiratory tract, the fact that influenza virus RNA and, in some cases, infectious influenza virus was detected and/or isolated from stools could indicate that the viruses were mixed with food and thus protected, or that the gastric acidity was reduced as a consequence of medical treatment [37], gastric disease [38], or mutations conferring resistance to low pH [39, 40].

### Remnants of infected submucosal intestinal antigen-presenting immune cells

The faecal presence of influenza virus RNA may be related to the detection of human influenza viral RNA in remnants of infected intestinal antigen-presenting immune cells. Influenza virus could bind intestinal cells such as DC-SIGN^+^ CD68^+^ dendritic cells, which are localized in the small and large intestine [41]. These intestinal DC-SIGN^+^ CD68^+^ cells act as antigen-presenting cells and participate in the stimulation of immunity through T-cell activation [42]. Antigen-presenting cells of various origins are susceptible to infection by different influenza virus subtypes [43], and may act as vehicles for extrapulmonary dissemination of the virus [44]. The shedding of viable influenza virus in stools in the absence of viraemia, suggests that the virus does not disseminate to the GI tract haematogenously after a primary respiratory tract infection [25, 45, 46].

### Replication in intestinal cells

Shu et al. [47] found that receptors for influenza virus were abundantly expressed on GI epithelial cells, which are highly permissive of influenza virus replication *in vitro* [48–50]. Human and avian influenza A viruses use different receptors for cell entry [51]. Human-adapted influenza A viruses preferentially bind to ‘humanlike’ sialic acid (SA)–α2,6–galactose (Gal)-terminated saccharides (hereafter, SA-α2,6-Gal), whereas avian influenza A viruses prefer receptors with ‘avian-like’ α2,3 linkages (hereafter, SA-α2,3-Gal). Examination of human colonic samples indicated that SA-α-2,6-Gal receptors are abundant on epithelial cells of the GI tract, and SA-α-2,3-Gal receptors can be found from the ileum to the rectum, with abundant expression of “avian-like” SA-α-2,3-Gal receptors in goblet cells being found mostly in the large intestine [47]. Both types of SA receptors are expressed on the surface of *in vitro* differentiated intestinal epithelial cells, suggesting that both avian and human influenza viruses have the potential to infect and replicate in human intestinal epithelial cells [47, 52]. Indeed, influenza A(H5N1) virus can directly target human gut tissues [47]. Intestinal epithelial cells are also susceptible to influenza A(H9N2) and A(H1N1)pdm09 viruses, and the infected cells become apoptotic with elevated pro-inflammatory responses [53, 54].

A mouse model of respiratory influenza infection was used to explore the hypothesis that respiratory influenza virus can enter the GI tract and as a direct consequence of its replication cause immune injury at this site [55]. Intranasal inoculation of the mice with the influenza A/PR/8/34 (PR8) strain led to injury within the intestine only when the virus infected the respiratory tract with immune injury occurring in the lung. In this model, no influenza virus was detected in the small intestine, and direct infection of the intestine with influenza virus did not lead to intestinal immune injury. The lymphocytes derived from the lung respiratory mucosa migrated into the intestinal mucosa during respiratory influenza infection via the CCL-25-CCR9 chemokine axis and destroyed the intestinal microbiota homeostasis in the small intestine, and the number of *Escherichia coli* (*E.coli*) in the intestinal tract increased, perhaps leading to intestinal immune injury. The hypothesis was Similar results were obtained when infecting mice with three different subtypes of respiratory influenza A viruses..

Influenza A virus significantly increases the adhesive properties of mucosa-associated *E. coli* strains, inducing the exposure of cellular receptors by intestinal cells [56]. The expression of these cellular receptors increased after influenza virus infection of lung epithelial cells [57], and influenza virus was shown to replicate efficiently in human primary intestinal cells. These findings suggest that viral infection of intestinal epithelial cells alters the glycosylation pattern of mucosal proteins and thereby increases bacterial adhesiveness, increasing the number of *E. coli*, thereby causing vomiting and diarrhea. These data suggest an increased number of *E. coli* as a consequence of influenza virus infection is the primary cause of intestinal injury during influenza virus infection.

## Conclusions

Although the human respiratory tract is the main target of infection by influenza viruses, whether human influenza viruses are capable of local GI replication is unclear. This systematic review and meta-analysis shows that the present knowledge on the clinical significance and pathophysiology of human influenza viruses in the GI tract is scarce.

The meta-analysis of the occurrence of GI symptoms among patients with influenza showed that they were inconsistent. First, the degree of heterogeneity among the ten studies included is so great that no specific GI symptoms can be described as typical for a patient with influenza. Therefore no comparison of the occurrence of GI symptoms among patients by types and/or subtypes of influenza viruses was possible. Second, the 95 % CIs of the prevalence rates were broad. The wide CIs could be related to the small number of studies included and to their small sample size. Moreover, the majority of studies used different criteria to define GI symptoms (either vomiting and/or diarrhea, abdominal pain or vomiting and diarrhea, or GI symptoms not specified), different laboratory methodologies (culture and/or RT-PCR, or serological tests) and were mostly conducted in hospitalized patients.

Similarly, studies reporting human cases with virological evidence of influenza virus in stools should be interpreted with caution because detection of viral RNA without additional virological evidence, such as culture or detection of anti-genomic RNA, does not necessary imply infection. Overall, in these studies, few clinical correlations were observed for viral RNA positivity and GI symptoms, and culture positivity was rare.

The source of influenza viruses in faeces and how the viruses pass through the GI tract is poorly understood. On the one hand, the presence of viral RNA in stools may be a consequence of haematogenous dissemination to organs through infected lymphocytes, while on the other hand, influenza viruses were able to increase the adhesive behaviour of mucosa-associated *E. coli* strains, inducing the exposure of cellular receptors through replication in intestinal cells.

Several methodological issues warrant discussion. The biggest challenge in extracting and compiling individual study data for this review was the variation in definitions of GI symptoms provided, the scarcity of data on the detection of RNA virus in stools, and the small sample size in the studies, providing little power for comparison between age classes or between types and subtypes of influenza viruses. Viral stool cultures from patients with respiratory infections (with and without GI symptoms) are infrequently required, and in the absence of standard methods for culturing influenza virus from stools, the true occurrence of infectious influenza virus in stools is unknown.

Additional studies of large prospective cohorts, examining GI symptoms in patients of all ages with severe and mild influenza, with systematic detection and isolation of influenza virus and other respiratory and enteric viruses from the upper respiratory tract and in stools concomitantly, and measuring influenza viral loads at respiratory and non-respiratory sites may provide further insights into the role of human influenza viruses in the GI tract. Understanding the viral shedding profiles of human influenza viruses might provide helpful information for understanding virulence, cell tropism, and transmission dynamics, and for designing management policies.

## Additional files

Additional file 1:
**Studies describing compared prevalence of gastrointestinal symptoms in function of influenza viruses.** (XLSX 11 kb) Additional file 2:
**Studies describing detection and/or isolation of influenza virus in stools of laboratory confirmed influenza patients.** (XLSX 12 kb)

## Abbreviations

GI: Gastrointestinal
CI: Confidence interval
*E.Coli*: *Escherichia coli.*
